# Usefulness of Laboratory-Based Machine Learning for Detection and Severity Classification of Acute Appendicitis in a Resource-Limited Healthcare Setting

**DOI:** 10.3390/diagnostics16071090

**Published:** 2026-04-04

**Authors:** Margarita L. Martinez-Fierro, Jose G. Gonzalez-Rodarte, Sodel Vazquez-Reyes, Manuel Gonzalez-Plascencia, Idalia Garza-Veloz, Perla Velasco-Elizondo, Sidere M. Zorrilla-Alfaro, Jaime Y. Burciaga-Paez, Gonzalo Ibarra-Bañuelos, Luis A. Flores-Chaires, Alejandro Mauricio-Gonzalez

**Affiliations:** Molecular Medicine Laboratory, Academic Unit of Human Medicine and Health Sciences, Universidad Autónoma de Zacatecas, Carretera Zacatecas-Guadalajara Km. 6, Ejido la Escondida, Zacatecas 98160, Mexico; margaritamf@uaz.edu.mx (M.L.M.-F.); 38192814@uaz.edu.mx (J.G.G.-R.); manuelgonzalezcharro@gmail.com (M.G.-P.); idaliagv@uaz.edu.mx (I.G.-V.); pvelasco@uaz.edu.mx (P.V.-E.); burciagapaezjy@gmail.com (J.Y.B.-P.); gonzaloib2997@gmail.com (G.I.-B.); luischaires@uaz.edu.mx (L.A.F.-C.)

**Keywords:** appendicitis, machine learning, support vector machines, clinical decision support systems, abdominal surgery

## Abstract

**Background**: Acute appendicitis is the most common abdominal surgical emergency, with diagnostic uncertainty greatest in resource-limited settings. **Objectives**: To develop and internally validate an interpretable, laboratory-driven machine learning approach to assist clinical decision-making in suspected appendicitis, including diagnosis, perforation detection, and surgical severity stratification. **Methods**: A retrospective cohort of 246 patients with histopathologically confirmed appendicitis and 45 controls with similar abdominal pain was analyzed at a secondary-level hospital in Mexico. After cleaning and imputation, 41 laboratory variables were used to train three models: Random Forest for appendicitis detection and perforation identification, and Support Vector Machine for surgical severity stratification. Class imbalance was addressed with synthetic oversampling, and feature selection prioritized clinical interpretability. **Results**: Appendicitis detection achieved excellent discrimination (AUC = 0.94), correctly identifying 90% of cases, with 100% specificity. The perforation model reached 100% sensitivity (AUC = 0.875), prioritizing safe detection of high-risk cases, while severity stratification showed moderate performance (AUC = 0.721), correctly identifying 81% of complicated cases without imaging. **Conclusions**: Laboratory-based ML models accurately detected acute appendicitis and identified all perforated cases using routine data alone, while surgical severity stratification showed moderate discrimination in the absence of imaging. These findings demonstrate the feasibility of laboratory-driven decision support for early risk assessment in resource-limited emergency settings and support further external validation.

## 1. Introduction

Worldwide, acute appendicitis remains the most common abdominal surgical emergency, representing a substantial burden on healthcare systems, particularly in low- and middle-income countries where timely access to advanced imaging modalities and specialized surgical care is limited [1,2,3]. The lifetime risk of developing appendicitis is estimated at 7–8%, with variations according to sex, geographic region, and healthcare availability [3,4]. Despite its long-standing recognition as a clinical entity, the diagnosis of acute appendicitis continues to pose significant challenges, especially in resource-limited settings where clinical assessment remains the primary diagnostic tool [1,5].

Clinical presentation is highly variable, ranging from the classic pattern of migrating right lower quadrant pain to atypical or nonspecific manifestations in pediatric patients, older adults, and pregnant women, substantially increasing the risk of diagnostic error [3,6,7]. This variability contributes directly to delayed diagnosis, unnecessary surgery, and preventable complications, emphasizing the need for improved diagnostic support in real-world clinical environments. In this context, clinicians face a persistent diagnostic trade-off: avoiding negative appendectomy and unnecessary surgical risk while preventing diagnostic delay, which increases the likelihood of progression to complicated appendicitis (perforation, abscess, or peritonitis) and is associated with higher morbidity, longer hospital stays, and increased mortality [5,6,8]. When decisions rely solely on clinical evaluation, negative appendectomy rates have historically ranged from 15% to 30%, highlighting the limitations of unaided clinical judgment [5,9].

Although the introduction of computed tomography (CT) and ultrasound has significantly improved diagnostic accuracy in high-complexity centers, these imaging modalities are not universally available due to cost, infrastructure, and personnel limitations, particularly in developing regions [5,7,9,10,11]. Consequently, many secondary-level hospitals must rely on incomplete diagnostic information, perpetuating a significant diagnostic gap and reinforcing the need for alternative, accessible decision-support strategies.

For decades, risk stratification has relied on linear clinical scoring systems such as the Alvarado score, the Pediatric Appendicitis Score (PAS), and the Appendicitis Inflammatory Response (AIR) score [12,13,14]. While clinically useful, these tools are inherently limited by their algorithmic rigidity and reduced performance outside derivation cohorts, as they cannot capture nonlinear interactions between physiological variables in heterogeneous populations [3,9,11].

Recent advances in artificial intelligence (AI) and machine learning (ML) offer a promising alternative. Unlike traditional statistical approaches, ML algorithms can model complex, high-dimensional relationships and have demonstrated potential to improve diagnostic accuracy, severity stratification, and complication prediction in appendicitis [7,9,11]. However, many previously proposed ML models rely heavily on imaging-derived features or complex feature engineering pipelines, which limits their feasibility in healthcare environments where advanced imaging infrastructure is unavailable or inconsistent [15,16,17]. Furthermore, several models operate as “black-box” systems with limited interpretability, reducing clinician trust and hindering translation into real-world clinical practice [11,15,18]. In addition, most previously published ML models have focused on a single prediction task, typically appendicitis diagnosis, while relatively few studies have attempted to address the sequential clinical decision-making process that occurs in real-world emergency settings, where clinicians must first determine the presence of appendicitis and subsequently evaluate disease severity and surgical risk [7,19].

This fragmentation highlights an important methodological and translational gap in the current literature. Moreover, although some models explore routine laboratory variables, few have simultaneously prioritized interpretability and clinical plausibility [15,16,18]. To our knowledge, a laboratory-based approach that simultaneously addresses multiple clinically relevant stages of appendicitis evaluation within a unified predictive framework is currently lacking.

In this context, the work by Zorrilla-Alfaro et al. [20] is particularly relevant, as it describes novel biomarkers derived from routine laboratory tests—the Inflammatory Metabolic Index (IMI) and the Metabolic–Inflammatory Stress Index (MISI), aimed at stratifying complicated and perforated appendicitis. Their findings suggest that readily available variables, such as glucose, creatinine, and white blood cell count, can be combined into composite indices with diagnostic and prognostic potential, highlighting the value of accessible laboratory parameters where advanced imaging is unavailable. Similarly, studies by Akbulut et al. [18] and Phan-Mai et al. [17] further substantiated the clinical relevance of metabolic and inflammatory indices by systematically assessing their diagnostic and prognostic performance and incorporating them into ML-based classification models. In these cohorts, derived indices such as NLR, WLR, WNR, and PNR consistently demonstrated discriminatory capacity for both diagnosis and severity stratification (perforated vs. non-perforated appendicitis) [17,18]. Their integration into supervised ML algorithms, including CatBoost and Gradient Boosting, improved overall classification performance and illustrated how routinely available laboratory parameters can be translated into interpretable ML-augmented decision-support tools, particularly in emergency and resource-constrained settings [17,18].

Building on these premises, the present study aimed to develop and internally validate an interpretable, laboratory-based ML pipeline designed to support three key clinical decisions point in suspected acute appendicitis: (1) initial diagnosis, (2) identification of perforated appendicitis, and (3) surgical severity stratification (complicated vs. uncomplicated appendicitis). Two Random Forest classifiers and one Support Vector Machine (SVM) were trained using exclusively routinely collected demographic and laboratory variables from a Mexican cohort, prioritizing interpretability and clinically guided feature selection over purely performance-driven optimization. The main contributions of this study can be summarized as follows:

1. Development of a laboratory-based ML pipeline relying exclusively on routinely available clinical and laboratory variables, facilitating potential implementation in healthcare environments lacking advanced imaging infrastructure.
2. Design of a sequential multi-task predictive framework that mirrors the real-world clinical decision-making process in suspected appendicitis, integrating diagnosis, perforation detection, and surgical severity stratification.
3. Implementation of clinically guided feature selection strategies prioritizing biological plausibility, stability, and interpretability of predictors.
4. Internal validation of the proposed models in a real-world Mexican cohort, demonstrating the feasibility of laboratory-driven ML decision support for acute appendicitis in resource-constrained settings.

## 2. Materials and Methods

### 2.1. Data Collection

This retrospective cohort study was conducted using a pre-existing clinical database derived from patients evaluated for suspected acute appendicitis at the Hospital General de Zacatecas Luz González Cosío, a secondary-level public hospital in Mexico, between July 2019 and May 2020 [20]. The database was originally constructed from routine clinical care records and subsequently structured for analytical use, serving as the sole data source for ML analyses. The study was explicitly designed as a feasibility and proof-of-concept investigation aimed at evaluating the clinical plausibility and discriminatory capacity of laboratory-based ML models in a resource-limited setting. Accordingly, the focus of the analysis was placed on model performance rather than on the interpretation of individual laboratory parameters. No feature-level hypothesis testing or biomarker discovery analyses were undertaken. The hospital provides emergency surgical care in an environment with limited access to advanced imaging modalities, where diagnostic decisions rely predominantly on clinical assessment and basic laboratory testing.

During the study period, a total of 382 patients with surgically evaluated acute abdominal conditions were initially eligible. From this cohort, 332 patients underwent appendectomy, irrespective of age or sex. General exclusion criteria applied to the entire database included previous appendectomy, pregnancy, and chronic or acute conditions known to alter inflammatory biomarkers (e.g., diabetes mellitus, chronic kidney disease, autoimmune diseases, active malignancies, or systemic infections), as well as the use of corticosteroids, immunosuppressants, or broad-spectrum antibiotics within seven days prior to admission.

Among the appendectomy cases, patients with incomplete laboratory or histopathological records and those undergoing incidental or secondary appendectomies were excluded (*n* = 86) to preserve diagnostic label integrity. The final analytical dataset consisted of 246 patients with histopathologically confirmed acute appendicitis, which constituted the primary population used for model development and internal validation.

**Figure 1:**
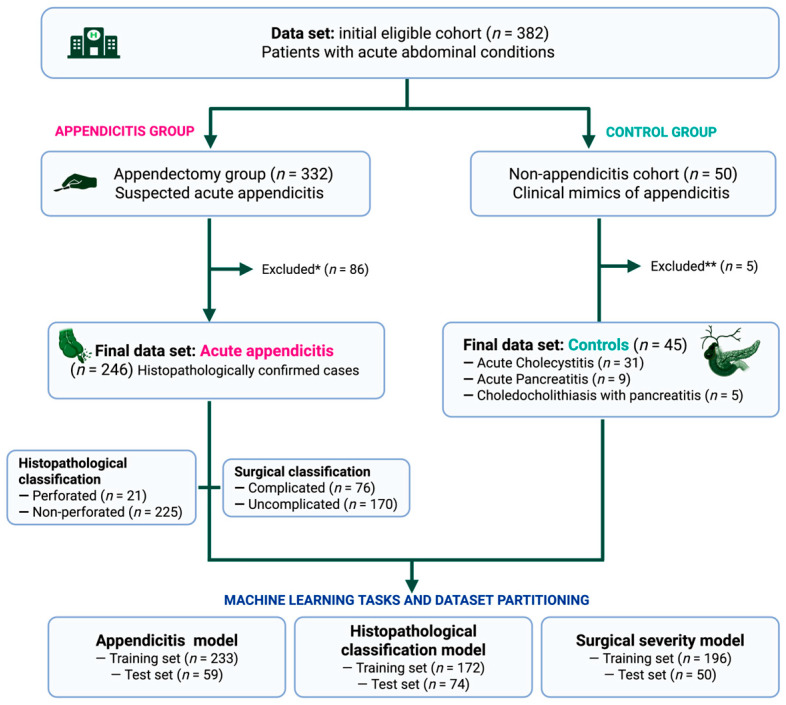
Flowchart of study population selection and dataset derivation. A data set with information of 382 patients with clinically suspected acute abdominal conditions was initially evaluated. Of these, 332 underwent appendectomy and were considered for the appendicitis group (ML development), while 50 patients with alternative diagnoses clinically mimicking appendicitis were considered as controls. In the appendicitis group, 86 patients were excluded (*) due to incomplete laboratory or histopathological records, incidental or secondary appendectomies, or predefined general exclusion criteria (including pregnancy, selected comorbidities, and recent treatments). This resulted in a final analytical dataset of 246 patients with histopathologically confirmed acute appendicitis used for model development and validation. These patients were further classified according to histopathological findings (perforated vs. non-perforated) and surgical severity (complicated vs. uncomplicated, according to the consensus guidelines of the European Association for Endoscopic Surgery). In the control group, 5 patients were excluded (**) due to incomplete clinical or laboratory data, yielding a final control dataset of 45 patients, including: acute cholecystitis (*n* = 31), acute pancreatitis (*n* = 9), and choledocholithiasis with pancreatitis (*n* = 5). For ML algorithms training and testing, three independent tasks were arranged: appendicitis diagnosis, histopathological classification, and surgical severity stratification. *n*, frequency.

In addition, 50 patients presenting with other acute abdominal conditions clinically mimicking appendicitis were initially identified as controls. A detailed flowchart outlining all inclusion and exclusion criteria, as well as the final number of patients assigned to each group, is presented in Figure 1.

After excluding five patients due to incomplete clinical or laboratory data, the final control dataset included 45 patients diagnosed with acute cholecystitis (*n* = 31), acute pancreatitis (*n* = 9), and choledocholithiasis with pancreatitis (*n* = 5). This control group was designed to represent a clinically relevant differential diagnosis population rather than a surgically selected cohort, thereby providing a realistic and challenging comparator for binary classification (appendicitis vs. non-appendicitis). Controls were used exclusively for appendicitis detection modeling and were not included in perforation or surgical severity analyses.

Confirmed appendicitis cases were further stratified according to two independent reference standards to support secondary modeling tasks. Perforated appendicitis (*n* = 21) was defined based on histopathological findings and represents a low-frequency but clinically high-impact outcome. Surgical severity was classified intraoperatively as complicated (*n* = 76; stages III–IV) or uncomplicated appendicitis (*n* = 170; stages I–II) in accordance with European Association for Endoscopic Surgery consensus guidelines.

Clinical and laboratory variables were extracted from medical records at emergency department admission. The initial dataset comprised 60 routinely collected features, including hematological indices (e.g., leukocyte and lymphocyte counts, neutrophil percentage, platelet indices), coagulation markers (prothrombin time, INR, partial thromboplastin time), and biochemical parameters (glucose, creatinine, and electrolytes such as sodium, potassium, chloride, phosphorus, and magnesium). To ensure model applicability at the point of care and minimize imputation bias, variables with more than 50% missing values were excluded, resulting in a final set of 39 features common to all models.

Overall, this dataset represents a clinically faithful snapshot of emergency surgical practice in a resource-limited setting and was deliberately constructed to evaluate feasibility, internal validity, and clinical plausibility rather than population-level generalization. This design aligns with the translational objective of the study and provides a robust foundation for future multicenter validation.

All experiments were implemented using the Python programming language (version 3.13.11), leveraging the Pandas, NumPy, and Scikit-learn libraries to ensure reproducibility and methodological consistency.

### 2.2. Dataset Cleaning

Incomplete laboratory testing across patients resulted in several features with a high proportion of missing values. To preserve data quality and minimize imputation-induced bias, variables with more than 50% missingness were excluded from further analysis, resulting in the removal of 18 features. This threshold was selected to balance feature retention with data reliability and to ensure that all retained variables were routinely available at emergency department admission, thereby preserving clinical applicability at the point of care. A complete description of the statistical distribution and missingness of all candidate laboratory variables prior to imputation is provided in Supplementary Table S1.

For the remaining variables, missing values were imputed using a K-Nearest Neighbors (KNN) multivariate imputation approach (*k* = 5). KNN was selected over model-based techniques such as Multiple Imputation by Chained Equations (MICE) because the objective of this study was to preserve local multivariate relationships between laboratory parameters rather than to generate multiple stochastic datasets for inferential analysis. In structured clinical datasets with moderate dimensionality, KNN imputation has been shown to better maintain correlation structure and distributional properties compared with univariate substitution or regression-based approaches, particularly when variables exhibit nonlinear relationships.

The number of neighbors was set to *k* = 5 to balance imputation stability and sensitivity to local structure: smaller *k* values increase variance and sensitivity to noise, whereas larger *k* values oversmooth clinically meaningful variability. This choice preserves physiologically plausible variation while avoiding distributional distortion of laboratory features, which is critical for downstream machine learning performance. KNN distance calculations were performed exclusively on the retained feature set after removal of variables with >50% missingness; the 14 excluded variables were therefore not involved in the imputation procedure. Distances between samples were computed using a NaN-aware Euclidean metric, in which pairwise distances are calculated using only the subset of variables observed in both samples. This approach prevents distortion of similarity estimates when partial laboratory panels are present and corresponds to the implementation of the KNNImputer algorithm in the Scikit-learn library.

All imputation procedures were performed using training data only to prevent information leakage. The test set was kept completely independent and was not used during model training, feature selection, or hyperparameter tuning.

Outlier values were reviewed manually by clinical criteria rather than statistical thresholds alone. Values within physiologically plausible ranges were retained, even when extreme, as they may reflect severe but clinically meaningful states (e.g., marked hypernatremia, leukocytosis, or coagulopathy). Conversely, values identified as data entry or measurement errors were corrected or excluded when deemed clinically implausible. This conservative strategy ensured that rare but high-impact laboratory abnormalities were preserved for model learning rather than inadvertently removed as noise. After dataset cleaning, the final analytical dataset comprised 246 confirmed appendicitis cases and 45 control cases, with 41 routinely available features shared across all models. This cleaning strategy prioritized internal validity, clinical realism, and model robustness, providing a reliable foundation for feasibility-focused ML evaluation in a resource-limited setting.

### 2.3. Data Preprocessing

To ensure compatibility with ML algorithms while preserving clinical interpretability, preprocessing procedures were systematically tailored to the type, scale, and clinical meaning of each variable. All preprocessing steps were performed exclusively on the training data and subsequently applied to the test sets to prevent information leakage and ensure unbiased performance estimation.

In addition, to ensure data quality and clinical reliability, all laboratory variables were obtained from standardized hospital measurement protocols within a controlled clinical environment. Prior to processing, a quality control step was performed in which variables with more than 50% missing values were excluded to reduce the impact of incomplete values. Additionally, extreme values were carefully reviewed manually based on clinical criteria to differentiate true pathological findings from potential measurement errors or data entry inconsistencies. These steps were implemented to minimize noise and improve the robustness of downstream ML models.

Categorical variables with more than two categories (e.g., blood type, surgical stump technique) were encoded using one-hot encoding, allowing the representation of discrete clinical states without imposing ordinal relationships. Multi-label binarization was applied only when variables could legitimately assume multiple simultaneous states, thereby avoiding artificial inflation of feature space. Binary categorical variables (e.g., sex) were encoded using binary encoding, preserving simplicity and interpretability.

Numerical variables were standardized using Z-score normalization, transforming each feature into a distribution with mean 0 and standard deviation 1:

$$Z=\frac{\left(x-\mu \right)}{\sigma }$$

where *x* represents the original value, *μ* the feature mean, and σ the standard deviation. Standardization was necessary to prevent features with larger numerical ranges (e.g., glucose, creatinine) from disproportionately influencing distance-based algorithms (KNN imputation, SVM) and tree-based split optimization, ensuring balanced model training across heterogeneous laboratory scales.

After encoding and normalization, three independent datasets were constructed corresponding to each clinical decision task, reflecting real-world diagnostic workflows rather than a single monolithic classification problem.

1. Appendicitis detection: appendicitis vs. non-appendicitis controls;
2. Histopathological classification: perforated vs. non-perforated appendicitis;
3. Surgical severity classification: complicated vs. uncomplicated appendicitis.

**Figure 2:**
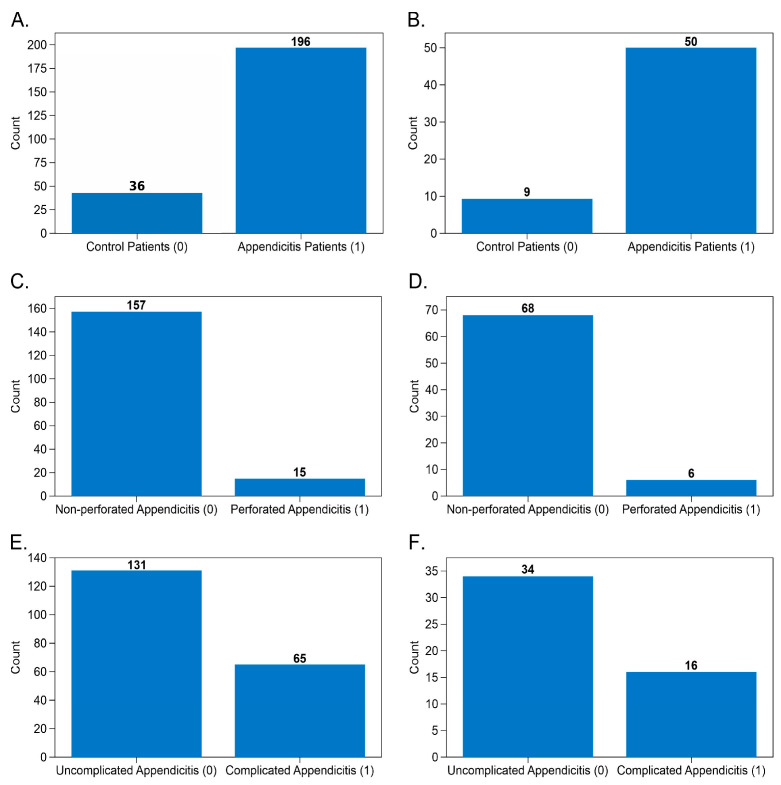
Class distribution across training and test sets for each classification task. Bar plots show the number of cases per class for each predictive task. Panels (**A**,**B**) display the distribution for the appendicitis detection model, with 196 appendicitis cases and 36 controls in the training set, and 50 appendicitis cases and 9 controls in the test set. Panels (**C**,**D**) show the histopathological classification (perforated vs. non-perforated), with 157 non-perforated and 15 perforated cases in training, and 68 non-perforated and 6 perforated cases in testing. Finally, panels (**E**,**F**) display distribution for the surgical severity stratification, with 131 uncomplicated and 65 complicated cases in training, and 34 uncomplicated and 16 complicated cases in testing.

Each dataset was partitioned into training and testing subsets using stratified sampling based on the outcome variable, ensuring that class proportions were preserved despite intrinsic imbalance. This step was critical given the low prevalence of perforated appendicitis and complicated disease, which represent rare but high-impact outcomes in emergency surgery. As illustrated in Figure 2A–F, all tasks exhibited varying degrees of class imbalance, particularly in the perforation and surgical severity datasets.

Instead of artificially equalizing distributions at this stage, the original prevalence was preserved to maintain clinical realism, and the imbalance was addressed during model training through appropriate resampling strategies. This approach avoids optimistic bias and ensures that model performance reflects real-world deployment conditions.

#### 2.3.1. Feature Selection

Feature selection was performed exclusively on the training set to prevent information leakage and optimistic bias during model evaluation. Importantly, this step was used as a regularization and interpretability strategy rather than as a means of performance maximization. The primary objective was to derive parsimonious models composed of a limited number of clinically interpretable predictors, thereby enhancing model robustness, reducing overfitting, and facilitating translational applicability in real-world clinical settings.

Rather than optimizing for maximal AUC or accuracy, feature selection prioritized clinical plausibility, stability across cross-validation folds, and consistency with known pathophysiological mechanisms of acute appendicitis. This approach intentionally sacrifices marginal performance gains to avoid feature fishing and to ensure that retained predictors represent reproducible biological signals rather than dataset-specific noise.

Feature selection was conducted independently for each predictive task, acknowledging that the biological processes underlying disease presence, tissue perforation, and surgical severity are related but not identical. This task-specific approach was motivated by clinical reasoning rather than algorithmic optimization.

Two complementary strategies were applied to evaluate feature relevance and stability: Forward selection and Backward selection.

Forward selection was implemented as an iterative wrapper procedure. Starting from an empty feature set, variables were sequentially added based on their contribution to cross-validated model performance (measured by AUC). At each iteration, the feature that produced the largest improvement in average cross-validated performance was retained until no further meaningful improvement was observed.

Backward selection was subsequently applied starting from the candidate feature set identified during forward selection. Features were removed one at a time while monitoring cross-validated performance, allowing identification of redundant predictors whose exclusion did not meaningfully reduce discriminative capacity. This complementary approach ensured that the final models remained both parsimonious and stable.

The combined forward–backward feature selection strategy was adopted to balance model performance and stability. Forward selection identifies variables that contribute meaningful predictive signal, while backward elimination removes redundant or weakly informative predictors that may introduce noise or increase model variance. This two-stage approach reduces the dimensionality of the feature space and mitigates overfitting, thereby improving model generalization when evaluated on unseen data.

#### 2.3.2. Final Feature Sets for Each Predictive Task

For the appendicitis detection task using a Random Forest (RF) classifier, forward selection yielded a concise set of laboratory predictors with clear clinical interpretability, including serum sodium, platelet count, mean corpuscular hemoglobin (MCH), mean platelet volume (MPV), international normalized ratio (INR), blood urea nitrogen (BUN), and neutrophil count. Rather than representing isolated markers, these variables span inflammatory activity, coagulation dynamics, intravascular volume status, and metabolic stress, providing a multidimensional yet parsimonious representation of early appendiceal inflammation. Backward selection demonstrated that removal of additional variables did not meaningfully reduce model discrimination, supporting the stability and sufficiency of this reduced feature set.

In the histopathological classification model (perforated vs. non-perforated appendicitis), the selected predictors shifted toward markers of systemic involvement and tissue-level injury, including MCV, RDW-CV, lymphocytes, segmented neutrophils, monocytes, PT, INR, glucose, creatinine, and potassium. This profile reflects progressive inflammatory burden, cellular stress, and early organ dysfunction, which are hallmarks of perforation rather than simple inflammation. Forward selection identified a minimal discriminative subset, while backward selection confirmed that the broader group of variables remained internally consistent and biologically aligned, reinforcing confidence in the robustness of the selected predictors.

For surgical severity stratification (complicated vs. uncomplicated appendicitis), feature selection using an SVM resulted in a broader and more heterogeneous predictor set, consistent with the multifactorial nature of surgical complexity. The retained features included hematological indices (hemoglobin, hematocrit, RDW-SD), inflammatory markers (total leukocyte count, band neutrophils), biochemical parameters (glucose, creatinine, potassium, sodium, chloride, phosphorus), and demographic variables (age, sex). Together, these variables capture the cumulative physiological impact of advanced disease, integrating inflammatory intensity, metabolic imbalance, fluid–electrolyte disturbances, and host susceptibility, thereby justifying the need for a wider feature representation in this task compared with binary diagnostic classification.

Across all three tasks, feature selection was guided by clinical coherence and stability across selection methods rather than by maximization of a single performance metric. This strategy prioritizes interpretability and regularization, ensuring that the final models function as robust decision-support tools grounded in biological plausibility, rather than as overfitted classifiers optimized for retrospective performance. The complete list of predictors retained for each modeling task is summarized in Supplementary Table S2.

#### 2.3.3. Model Training and Evaluation

**Figure 3:**
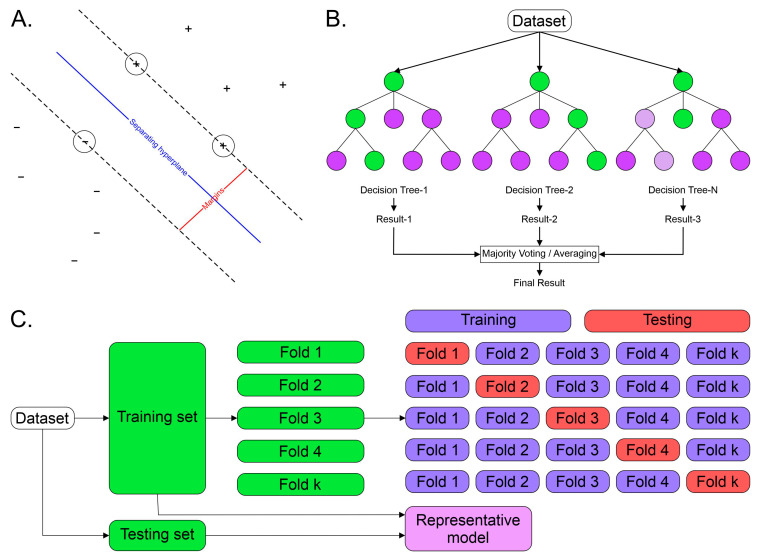
Overview of machine learning algorithms and evaluation strategy used in this study. Panel (**A**) depicts the Support Vector Machine (SVM) classifier, showing the optimal separating hyperplane, the margin, and the support vectors that define the boundary. Panel (**B**) illustrates the Random Forest architecture, where multiple decision trees are trained on bootstrapped samples and random feature subsets. Each tree produces a prediction, and the final output is determined by majority voting or averaging. Panel (**C**) presents the *k*-fold cross-validation process, where the training set is partitioned into five folds. Each fold is used once as a validation set, ensuring robust estimation of bias and variance, and supporting hyperparameter tuning.

Rather than optimizing a single algorithm for maximal retrospective performance, three complementary supervised learning models were deliberately developed to reflect the sequential clinical reasoning used in emergency evaluation of suspected acute appendicitis. This design mirrors the stepwise decision-making process of clinicians, moving from initial diagnosis to severity assessment, and is visually summarized in Figure 3.

Three classifiers were implemented, each aligned with a specific clinical decision point: (i) an RF model for appendicitis detection, (ii) a second RF model for identification of perforated appendicitis, and (iii) an SVM model for surgical severity stratification (complicated vs. uncomplicated appendicitis). This architecture prioritizes clinical relevance and interpretability over algorithmic uniformity, acknowledging that different diagnostic stages require different inductive biases.

As illustrated in Figure 3A, the SVM model constructs an optimal separating hyperplane that maximizes the margin between classes, focusing learning on critical borderline cases represented by support vectors. This property is particularly advantageous for surgical severity stratification, where physiological deterioration occurs along a continuum rather than as a binary shift. A radial basis function (RBF) kernel was used to enable nonlinear separation while preserving generalization in moderate-sized clinical datasets.

For appendicitis detection and perforation identification, RF classifiers were selected due to their robustness to noise, ability to model nonlinear interactions, and stability in heterogeneous clinical data. As shown in Figure 3B, the RF algorithm aggregates predictions from multiple decorrelated decision trees trained on bootstrapped samples and random feature subsets, with the final output determined by majority voting. This ensemble structure reduces variance and mitigates overfitting, making it particularly suitable for real-world surgical cohorts with mixed laboratory and demographic features.

Class imbalance, especially pronounced in perforated and complicated appendicitis, was addressed using synthetic minority oversampling applied exclusively to the training set, thereby preventing information leakage. Synthetic samples were generated through interpolation in feature space between minority-class instances, enriching the decision boundary without duplicating observations and improving model sensitivity to rare but clinically high-impact outcomes.

Model generalizability was assessed using 5-fold cross-validation, following the scheme depicted in Figure 3C. The dataset was first split into independent training and testing sets. The training set was then partitioned into five stratified folds, with each fold used once for validation while the remaining folds were used for training. This procedure reduces variance associated with single splits, provides robust estimates of model stability, and supports conservative hyperparameter tuning. The final representative model for each task was selected based on average cross-validated performance rather than peak results in any single fold, reinforcing robustness over optimism.

Model evaluation focused on clinically meaningful performance metrics. Discriminative ability was primarily quantified using the receiver operating characteristic (ROC) curve and the area under the curve (AUC), which reflect performance across all decision thresholds. Given the clinical consequences of delayed diagnosis or underestimation of severity, additional metrics derived from the confusion matrix, including sensitivity, specificity, and precision, were reported to explicitly balance false-negative and false-positive errors.

## 3. Results

The final analytical dataset comprised 246 patients (51.6% male, mean age 24.8 ± 19.3 years), of which 196 had histopathologically confirmed acute appendicitis. In addition, a control dataset of 45 patients with acute abdominal conditions was included, comprising cases of acute cholecystitis (*n* = 31), acute pancreatitis (*n* = 9), and choledocholithiasis with pancreatitis (*n* = 5).

Decision thresholds for each model were selected based on a combination of ROC analysis and clinical prioritization criteria. Specifically, thresholds were chosen to balance sensitivity and specificity while prioritizing sensitivity in clinically critical tasks (e.g., perforation detection), where false negatives carry higher clinical risk. This approach aligns with real-world emergency decision-making, where early identification of high-risk patients is essential.

### 3.1. Appendicitis Detection Model

**Figure 4:**
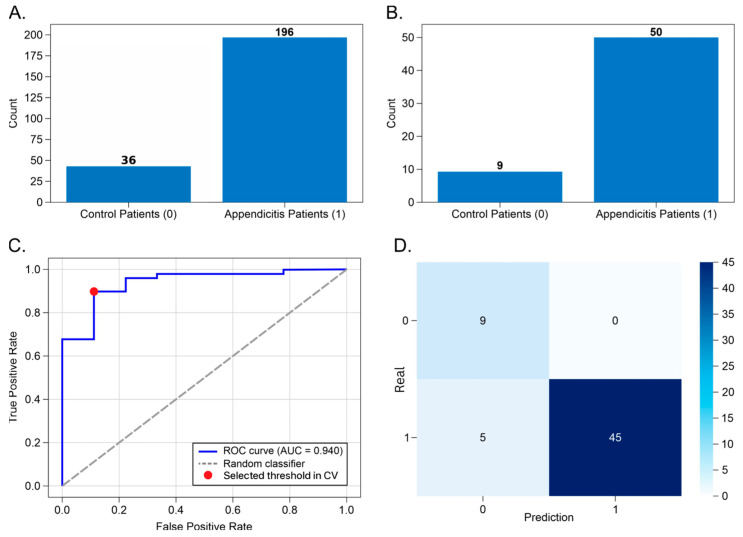
Performance summary of the appendicitis detection model using Random Forest classifier. Panel (**A**) shows the class distribution in the training set, with 196 appendicitis cases and 36 control cases. Panel (**B**) displays the test set distribution, comprising 50 appendicitis cases and 9 controls. Panel (**C**) presents the ROC curve obtained during cross-validation for internal model development and threshold selection, with an AUC of 0.94, indicating high discriminative capability. Panel (**D**) shows the confusion matrix at a decision threshold of 0.61: all control cases were correctly classified (true negatives = 9), while five appendicitis cases were misclassified (false negatives = 5), yielding a sensitivity of 0.90 and precision of 1.00.

The appendicitis detection task was addressed using an RF classifier trained to discriminate patients with acute appendicitis from controls presenting with clinically similar abdominal conditions. The dataset was partitioned into a training set (80%) and an independent test set (20%), maintaining class proportions. As shown in Figure 4A,B, the training set included 196 appendicitis cases and 37 controls, while the test set comprised 50 appendicitis cases and 9 controls, reflecting the natural imbalance observed in real-world surgical cohorts.

When evaluated on the independent test set, the model demonstrated excellent discriminative ability, achieving an AUC of 0.94, meaning that 94% of patients with appendicitis were correctly ranked above non-appendicitis controls using laboratory data alone (Figure 4C). At the predefined decision threshold (0.61), the model correctly identified 90% of patients with appendicitis, yielding a sensitivity of 0.90, while all control patients were correctly classified, resulting in a specificity and precision of 1.00 (Figure 4D, Table 1).

Clinically, this performance indicates that the model functions as a high-confidence diagnostic filter, capable of excluding non-appendicitis cases while preserving high sensitivity for true disease. The five misclassified appendicitis cases represent early or atypical presentations, consistent with the known limitations of laboratory-only assessment.

Feature selection identified a compact and clinically coherent predictor set: serum sodium, platelet count, MCH, MPV, INR, BUN, and neutrophil count, capturing complementary physiological domains including inflammation, coagulation activation, intravascular volume status, and metabolic stress. Together, these results demonstrate that clinically meaningful diagnostic discrimination can be achieved without reliance on imaging, approaching the performance of CT-based strategies reported in high-resource settings.

**Figure 5:**
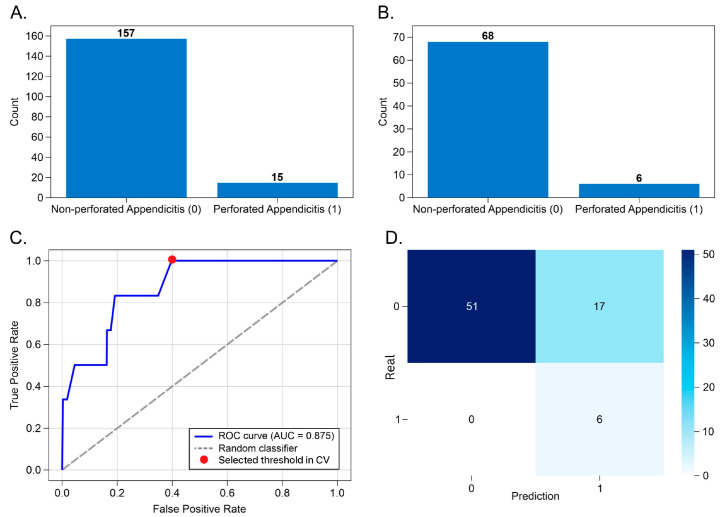
Performance summary of the histopathological diagnosis model using Random Forest classifier. Panel (**A**) shows the class distribution in the test set, with 68 non-perforated and 6 perforated appendicitis cases. Panel (**B**) displays the training set distribution, comprising 157 non-perforated and 15 perforated cases. Panel (**C**) presents the ROC curve obtained during cross-validation for internal model development and threshold selection, with an AUC of 0.875, indicating strong discriminative performance. Panel (**D**) shows the confusion matrix at a decision threshold of 0.58: all perforated cases were correctly classified (true positives = 6), while 17 non-perforated cases were misclassified (false positives), yielding a sensitivity of 1.00 and precision of 0.26.

**Figure 6:**
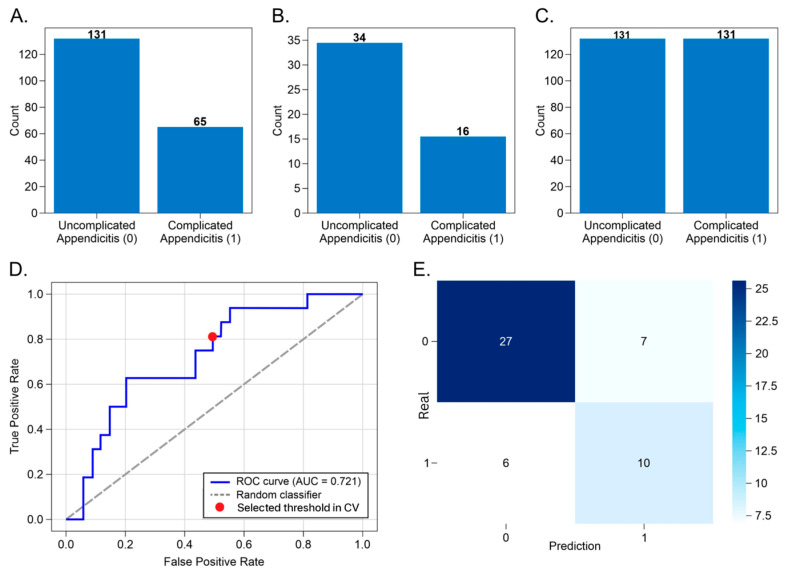
Performance summary of the surgical diagnosis model using Support Vector Machine classifier. Panel (**A**) shows the class distribution in the original training set, with 131 uncomplicated and 65 complicated appendicitis cases. Panel (**B**) displays the test set distribution, comprising 34 uncomplicated and 16 complicated cases. Panel (**C**) illustrates the balanced training set after synthetic oversampling, with 131 instances per class. Panel (**D**) presents the ROC curve obtained during cross-validation for internal model development and threshold selection, with an AUC of 0.70, indicating moderate discriminative performance. Panel (**E**) shows the confusion matrix at a decision threshold of 0.36: 10 complicated cases were correctly classified (true positives), while 7 uncomplicated cases were misclassified (false positives), yielding a sensitivity of 0.81 and precision of 0.56.

It is important to note that the ROC curves in Figure 4C, Figure 5C, and Figure 6D were derived during the cross-validation phase used for internal model development and threshold selection. In each case, the highlighted red marker indicates the threshold selected during cross-validation, rather than the exact sensitivity/specificity pair observed on the independent test set.

In contrast, the sensitivity, specificity, and precision values reported in Table 1, Table 2 and Table 3 correspond to the models’ observed performance on the independent test sets after applying these predefined thresholds. Because threshold selection was intentionally performed prior to test-set evaluation to avoid post hoc optimization, the exact test-set operating coordinates do not necessarily coincide visually with the points highlighted on the ROC curves.

### 3.2. Histopathological Diagnosis Model

The second task focused on identifying perforated appendicitis, a low-prevalence but high-impact outcome, using an RF classifier trained exclusively on laboratory variables. The training set included 15 perforated and 157 non-perforated cases, while the test set comprised 6 perforated and 68 non-perforated cases, reflecting the expected real-world prevalence of perforation (Figure 5A,B).

On the test set, the model achieved an AUC of 0.875, indicating strong discriminative capacity for this rare but clinically critical outcome (Figure 5C). At the selected decision threshold (0.58), the model achieved 100% sensitivity, correctly identifying all perforated cases, while specificity was 0.75 (Figure 5D, Table 2). As expected for a model optimized for rare-event detection, precision was 0.26, reflecting deliberate over-triage.

Importantly, this performance profile represents a clinically desirable trade-off: the model prioritizes sensitivity over precision, ensuring that no perforated appendices are missed, at the cost of labeling some non-perforated cases as high risk. In surgical practice, this behavior is appropriate for a rule-out safety tool, where the harm of missed perforation far exceeds the cost of additional vigilance.

The low precision is expected given the extreme class imbalance and the low prevalence of perforated appendicitis in the test set. Precision is known to be highly sensitive to class prevalence and may therefore underestimate model utility in rare but clinically critical conditions. Furthermore, the achieved ROC-AUC of 0.875 indicates strong discriminative capability independent of decision threshold, supporting the overall robustness of the model.

Feature selection retained laboratory markers associated with systemic inflammatory burden and early organ dysfunction, including MCV, RDW-CV, lymphocytes, segmented neutrophils, monocytes, PT, INR, glucose, creatinine, and potassium, supporting the biological plausibility of the learned decision patterns. These results indicate that perforation risk can be reliably flagged using laboratory data alone, even in the absence of imaging.

### 3.3. Surgical Diagnosis Model

The final task addressed differentiation between complicated and uncomplicated appendicitis using an SVM classifier, reflecting the most clinically complex and heterogeneous decision point. The original training set included 131 uncomplicated and 65 complicated cases, while the test set comprised 34 uncomplicated and 16 complicated cases (Figure 6A,B). To mitigate class imbalance, synthetic minority oversampling was applied exclusively to the training set, resulting in a balanced distribution (Figure 6C).

On the independent test set, the SVM model achieved an AUC of 0.721, a level of performance that is expected in the absence of imaging variables and reflects the intrinsic difficulty of surgical severity stratification using laboratory data alone (Figure 6D). Despite this, the model correctly identified 10 of 16 complicated cases, yielding a sensitivity of 0.81, while maintaining a specificity of 0.70 and a precision of 0.56 (Figure 6E, Table 3).

These results indicate that the model provides a clinically meaningful baseline for early severity stratification, outperforming unstructured clinical judgment alone and offering objective support in resource-limited settings where imaging is unavailable or delayed. The broader feature set retained for this task, including hematological indices, inflammatory markers, biochemical parameters, and demographic variables, reflects the cumulative physiological derangement associated with advanced disease and explains the need for a more complex decision boundary.

The three models demonstrate progressively decreasing but clinically appropriate performance across increasingly complex decision stages, supporting their use as complementary decision-support tools rather than standalone diagnostic systems.

### 3.4. Model Interpretability and Clinically Relevant Predictors

The models also provided clinically interpretable insights by identifying laboratory parameters that contributed most strongly to classification decisions. For appendicitis detection, neutrophil count, serum sodium, platelet count, mean platelet volume, BUN, INR, and MCH emerged as the most informative predictors. These variables represent complementary physiological domains including inflammatory activation, coagulation changes, intravascular volume status, and metabolic stress, which are well-recognized biological responses during acute appendiceal inflammation.

In the perforation detection model, markers associated with systemic inflammatory burden and tissue injury, such as RDW-CV, MCV, lymphocyte and monocyte counts, PT/INR, glucose, creatinine, and potassium, contributed most strongly to prediction, suggesting that the model captured patterns consistent with progressive systemic involvement and early organ dysfunction.

For surgical severity stratification, the SVM model relied on a broader set of hematological indices, inflammatory markers, biochemical parameters, and demographic variables, reflecting the multifactorial physiological alterations associated with complicated appendicitis. Together, these findings support the biological plausibility of the models and demonstrate that machine learning can identify clinically meaningful predictors from routine laboratory data.

## 4. Discussion

Acute appendicitis remains a significant diagnostic challenge, particularly in settings where access to advanced imaging is limited [21]. Although CT has become the diagnostic gold standard in high-complexity hospitals when clinical uncertainty exists, this approach is not universally applicable, especially in low- and middle-income countries [6,10,17]. In this context, the use of ML models based on routinely available clinical and laboratory data has emerged as a promising alternative to support decision-making, provided that their performance is interpreted within the appropriate clinical and methodological context [19].

The results of the present study suggest that tabular ML models trained on routine variables can achieve discriminative performance comparable to that reported in international studies. The appendicitis detection model achieved an AUC of 0.94, which lies within the range described for models based on RF, Gradient Boosting, and advanced logistic regression (AUC 0.89–0.95), and exceeds the performance of traditional clinical scores such as Alvarado, PAS, and AIR [16,18,22,23]. Reported diagnostic performance for these traditional scores typically ranges between AUC values of approximately 0.70–0.82 [1,7]. The superior performance observed in our model may be explained by the ability of ML algorithms to capture nonlinear interactions between laboratory parameters that are not accounted for in additive scoring systems [22,23,24]. Importantly, ML-based approaches should not be interpreted as replacements for established scores, but rather as complementary decision-support tools that could refine risk stratification in settings where imaging resources are limited [7,15,22].

Nevertheless, given the sample size and the single-center nature of the dataset, these findings should be interpreted as evidence of feasibility rather than as proof of superiority.

Dataset quality represents a critical factor in the development and evaluation of clinical ML models. In this study, several steps were performed to ensure data reliability, including the exclusion of variables with high missingness, clinically guided review of extreme values, and the application of standardized laboratory measurements obtained within a single institutional setting. These measures aimed to reduce noise and improve the consistency of the input data.

However, despite these precautions, the retrospective design and single-center origin of the dataset may introduce inherent source bias, including selection bias and limited variability in laboratory recollection protocols. Additionally, the use of imputation methods, while necessary to preserve sample size, may introduce uncertainty in variables with complete observations. Consequently, although the models demonstrated strong internal performance, their generalizability to other clinical settings with different populations or laboratory standards remains uncertain and should be confirmed through external validation.

An additional methodological consideration concerns the choice of the ML algorithms used in this study. In structured tabular clinical datasets of moderate size, classical machine learning algorithms frequently outperform more complex approaches such as deep learning models, which generally require substantially larger datasets to achieve stable generalization [7,25]. RF and SVM were selected because both methods have demonstrated strong and stable performance in these types of datasets.

RF is well suited for modeling nonlinear interactions and heterogeneous biomedical variables while maintaining robustness against overfitting. In contrast, SVM models are particularly effective in identifying complex decision boundaries by constructing optimal separating hyperplanes. For this reason, several recent comparative studies in clinical prediction modeling report that algorithms such as RF and SVM often provide performance comparable to or better than more computationally intensive approaches when applied to routine clinical data [25,26,27,28].

From a pathophysiological perspective, the predictors selected by the model (neutrophil count, sodium, MPV, platelet count, BUN, and INR) reflect mechanisms widely described in the literature, including systemic inflammation, metabolic stress, electrolyte imbalance, and coagulation activation [9,20,22,24]. Recent work by Zorrilla-Alfaro et al. [20] formalized these interactions using composite indices such as the IMI and MISI, which showed improved performance in identifying complicated and perforated appendicitis compared with classic markers such as the NLR. In contrast to that approach, the present study extends this line of work by training tabular ML models directly on routinely collected clinical and laboratory variables and organizing them into a stepwise, interpretable decision-support pipeline aligned with emergency workflows. While prior studies focused on the performance of individual indices or isolated classification tasks, our approach addresses multiple sequential clinical decisions, including diagnosis, perforation detection, and surgical severity stratification.

An additional advantage of the present approach lies in model interpretability. The variables prioritized by the algorithms correspond to laboratory parameters with well-established roles in acute inflammatory processes and systemic physiological stress. Neutrophilia reflects innate immune activation, platelet indices capture inflammatory–coagulation interactions, while electrolyte and metabolic markers may indicate dehydration and systemic stress associated with acute abdominal inflammation. The fact that these biologically plausible variables consistently emerged as key predictors suggests that the models capture meaningful clinical patterns rather than purely statistical correlations. Such interpretability is essential for clinical implementation, as decision-support systems are more likely to be adopted when their predictions can be understood and justified within established medical knowledge.

The histopathological classification model showed high sensitivity for detecting perforated appendicitis, a pattern consistently reported in studies with highly imbalanced classes [24]. Research using RF or XGBoost models has described similar behavior, in which optimization for rare events leads to reduced overall accuracy.

Although the precision of the perforation model was low, this behavior reflects a deliberate and clinically appropriate trade-off. In emergency surgery, the clinical cost of a false-negative perforation (delayed intervention, peritonitis, and increased morbidity) far exceeds the cost of false-positive alerts, which typically result in increased monitoring, expedited imaging, or earlier surgical consultation rather than unnecessary intervention [24]. From this perspective, the model functions as a safety-oriented rule-out tool rather than a balanced classifier, aligning with established triage principles in emergency medicine where sensitivity is intentionally prioritized for high-risk outcomes.

In contrast, the moderate performance of the surgical classification model aligns with previous reports indicating that distinguishing between complicated and uncomplicated appendicitis is particularly challenging when only structured clinical data are available. Studies reporting higher AUCs typically incorporate imaging variables (e.g., appendiceal diameter, fecalith, free fluid) or use multimodal approaches [7]. This finding reinforces the notion that laboratory data alone may be sufficient to support initial diagnosis but remains limited for detailed surgical stratification, particularly in the absence of ultrasound or CT imaging.

From a methodological perspective, the performance observed across the three prediction tasks reflects the increasing complexity of the underlying clinical decisions. The appendicitis detection model achieved the highest discriminative performance, which is consistent with the fact that early inflammatory changes produce measurable alterations in routine laboratory parameters. In contrast, perforation detection and surgical severity classification represent later and more heterogeneous disease stages, which inherently introduce greater biological variability and therefore reduce achievable predictive accuracy. The statistical evaluation performed using independent test sets and ROC-based metrics supports the robustness of these findings and suggests that the models capture reproducible clinical patterns rather than dataset-specific correlations. Importantly, these results should be interpreted as evidence of analytical feasibility rather than definitive proof of diagnostic superiority, highlighting the need for prospective validation in larger multicenter cohorts.

In line with this observation, recent international studies have reported similar limitations and strengths of laboratory-based ML models across different clinical settings. A comparative summary of these studies, including model types, input variables, and reported performance metrics, is provided in Table 4.

An important contribution of this work is its alignment with stepwise diagnostic strategies proposed in the literature, in which laboratory-based ML models are used as an initial filter to reduce unnecessary advanced imaging [9,22,29]. Economic and clinical studies have shown that such strategies can reduce radiation exposure, healthcare costs, and negative appendectomy rates without compromising patient safety [17,21,30]. In this context, our findings support the potential integration of tabular ML models into pragmatic diagnostic protocols for secondary-level hospitals.

From a clinical perspective, laboratory-based ML models could be integrated as early decision-support tools within electronic medical record systems or lightweight clinical applications, allowing automated risk estimation immediately after laboratory results become available. Such tools may assist clinicians in prioritizing imaging studies, identifying high-risk patients requiring early surgical evaluation, and reducing diagnostic uncertainty in emergency departments. These advantages may be particularly relevant in resource-limited hospitals, where imaging availability is restricted and rapid risk stratification based on routinely available laboratory data could improve both diagnostic efficiency and patient safety.

From a health systems perspective, laboratory-based ML models may also offer economic advantages. Because the models rely exclusively on routinely collected laboratory parameters, their implementation would not require additional diagnostic testing or infrastructure beyond standard hospital information systems. In resource-limited settings, this approach could help prioritize imaging for high-risk patients while safely reducing unnecessary CT utilization, potentially lowering diagnostic costs and radiation exposure.

Furthermore, the ability of the models to identify patients without evidence of systemic complications suggests a possible role in selecting candidates for conservative antibiotic management, a strategy that has been shown to be safe in uncomplicated appendicitis but relies critically on accurate early stratification [24]. However, this application should be prospectively evaluated before any clinical implementation [16,18].

Finally, the recent literature highlights the importance of model interpretability for successful clinical adoption [11,15,18]. Although this study prioritized intrinsically interpretable models and feature selection, formal explainability analyses were not performed. Future work incorporating methods such as SHAP could help validate that model predictions are aligned with established pathophysiological mechanisms, thereby facilitating acceptance among surgeons and emergency physicians [7].

Our findings, when interpreted in the context of existing evidence, suggest that ML models based on structured clinical data may serve as useful decision-support tools for the early detection of appendicitis in resource-limited settings. Nevertheless, definitive clinical utility will depend on multicenter validation, prospective evaluation, and careful integration into established clinical workflows [7,16,22].

### 4.1. Limitations

This study should be interpreted as a feasibility and translational investigation rather than a definitive diagnostic solution. Its primary contribution lies in demonstrating that routinely collected clinical and laboratory data can be leveraged to train interpretable machine learning models capable of supporting key clinical decisions in suspected acute appendicitis, particularly in resource-limited environments. As such, the findings provide evidence of clinical plausibility rather than claims of immediate clinical utility as a replacement for clinical judgment or universal generalizability.

An important limitation concerns the size and composition of the control group, which was substantially smaller than the appendicitis cohort (45 controls vs. 246 cases). This imbalance reflects the inherent structure of surgical diagnostic pathways, in which histopathological confirmation is available only for operated patients, while most patients with non-appendicitis abdominal pain are managed conservatively and therefore do not generate definitive ground truth. As a result, the control group represents a clinically challenging subset enriched for diagnostic uncertainty rather than a population-based reference, which may limit generalizability to unselected emergency department populations. This workflow introduces verification bias, a well-recognized and largely unavoidable limitation in surgical datasets, reinforcing the need for multicenter validation incorporating non-operated controls with standardized follow-up. Furthermore, the single-center design reflects a deliberate focus on data consistency, diagnostic fidelity, and standardized laboratory protocols, which strengthens internal validity but limits population diversity. Differences in patient demographics, clinical workflows, and laboratory reference ranges across hospitals may influence model performance. Therefore, the generalizability of laboratory-based appendicitis prediction models across different healthcare systems remains an open question that requires validation using independent external datasets.

Class imbalance, particularly for perforated and complicated appendicitis, posed a methodological challenge. However, the limited number of such cases accurately reflects real-world prevalence and highlights the difficulty of modeling rare but clinically high-impact outcomes. Although synthetic oversampling improved training stability and sensitivity, performance estimates for these subgroups remain inherently sensitive to small variations in case numbers, especially in independent test sets. Larger and more heterogeneous datasets are therefore required to strengthen decision boundaries for rare clinical events.

Another limitation is the exclusive reliance on clinical and laboratory data without imaging variables. While this approach intentionally enhances interpretability, accessibility, and applicability in low-resource settings, it restricts direct comparison with image-based deep learning models and limits achievable performance in advanced severity stratification. Nevertheless, this design choice reflects the clinical reality of many secondary-level hospitals, reinforcing their translational relevance.

Finally, although feature selection provided insights into biologically plausible predictors, formal explainability analyses were not performed. Dedicated explainability frameworks such as SHAP-based feature attribution could provide deeper insight into model decision mechanisms and further strengthen interpretability, transparency, and clinical trust.

### 4.2. Future Directions

Future research should prioritize multicenter prospective validation, addressing the most persistent limitation of this study: restricted sample size and single-institution data. Validation across multiple hospitals with diverse patient populations, including pediatric and elderly cohorts, will allow evaluation of model robustness under heterogeneous clinical conditions and laboratory protocols. Such multicenter datasets would also enable external validation on independent populations, which represents a critical step toward establishing true clinical generalizability.

Expansion of the dataset would additionally improve modeling of rare outcomes such as perforated appendicitis. Future studies should explore advanced strategies for class imbalance management, including cost-sensitive learning, prevalence-aware threshold calibration, and simulation of real-world disease prevalence. These approaches may further optimize sensitivity for clinically critical outcomes while maintaining acceptable specificity.

With larger and more diverse datasets, model recalibration and systematic hyperparameter optimization will be essential. Comparative evaluation of alternative interpretable algorithms, such as gradient boosting machines, regularized logistic regression, or hybrid ensemble models, should be performed while maintaining the study’s emphasis on reproducibility and clinical interpretability.

Another important direction involves the integration of explainable artificial intelligence techniques. Global and local interpretability tools, particularly SHAP-based feature attribution, could provide transparent visualization of variable contributions and facilitate clinical understanding of model behavior. Such explainability frameworks are increasingly required for regulatory approval and clinical implementation of ML-based decision-support systems.

Future work should also explore multimodal predictive strategies. Hybrid models that combine laboratory variables with optional imaging features, when available, may improve diagnostic accuracy while maintaining the flexibility required for resource-limited environments where imaging access is inconsistent.

In addition to methodological improvements, clinical integration studies are required. In the same sense, prospective pilot implementation as a clinical decision-support tool, potentially through a lightweight mobile or web-based application, would allow evaluation of impact on diagnostic efficiency, time to surgical decision-making, imaging utilization, healthcare costs, and patient outcomes. Such translational studies are essential to determine whether ML-assisted decision support can meaningfully improve clinical workflows in emergency and surgical settings.

Ultimately, the combination of multicenter validation, advanced interpretability frameworks, and prospective clinical deployment studies will be necessary to establish the role of laboratory-based machine learning models as scalable decision-support tools for the management of acute appendicitis.

## 5. Conclusions

This study demonstrates that interpretable ML models trained exclusively on routinely available clinical and laboratory variables can provide meaningful decision support for acute appendicitis in resource-limited settings. For appendicitis detection, the RF model achieved an AUC of 0.94, with 90% sensitivity, 100% specificity, and precision on the independent test set, indicating that laboratory-only data can reliably function as a high-confidence diagnostic filter in patients presenting with abdominal pain.

For perforation identification, the second RF model reached an AUC of 0.875 and, importantly, achieved 100% sensitivity with 75% specificity, reflecting a clinically desirable over-triage profile for this low prevalence but high-risk outcome. This performance ensures that no perforated cases are missed, supporting its role as a rule-out safety mechanism when imaging is unavailable or delayed.

Although surgical severity stratification remains intrinsically challenging without imaging, the SVM model achieved an AUC of 0.721, with 81% sensitivity and 70% specificity, correctly identifying most complicated cases. This level of performance reflects the inherent complexity of this decision point and provides objective support superior to unaided clinical assessment in resource-constrained environments.

Together, our results offer a viable alternative to reduce diagnostic uncertainty, optimize imaging utilization, and support timely intervention. Rather than proposing a definitive diagnostic replacement, these findings support future multicenter validation and progressive integration into care pathways aimed at improving equity, efficiency, and safety in surgical care.

## Figures and Tables

**Table 1:** Appendicitis model metrics.

AUC	Sensitivity	Specificity	Precision
0.94	0.9	1.0	1.0

**Table 2:** Histopathologic diagnosis model metrics.

AUC	Sensitivity	Specificity	Precision
0.875	1.0	0.75	0.26

**Table 3:** Surgical diagnosis model metrics.

AUC	Sensitivity	Specificity	Precision
0.721	0.81	0.70	0.56

**Table 4:** Summary of recent machine learning studies for the diagnosis and stratification of acute appendicitis using structured clinical and laboratory data.

Study (Author, Year, Country)/Reference	Population (*n*) and Study Design	ML Model Used	Main Input Variables	Clinical Applicability	Reported Performance (Validation/Test)
Aydin et al. [16] (2025) Türkiye	*n* = 11,622 (8586 training + 3036 external validation) Pediatric Prospective, multicenter	Random Forest (Outperformed SVM, KNN, LR, and traditional scales like PAS/Alvarado)	Clinical data and complete blood count (WBC, Neutrophils, MPV, RDW, Platelets). No imaging.	Primary diagnosis in pediatric emergencies without the need for radiation (CT). Robust external validity.	AUC: 0.996 Sensitivity: 99.8% Specificity: 99.3% Accuracy: 99.2%
Kucukakcali et al. [15] (2025) Türkiye	*n* = 590 pediatric patients. Retrospective observational study.	Random Forest (RF) and XGBoost (compared to AdaBoost, SGB, Bagged CART)	CRP, white blood cell (WBC) count, neutrophils, lymphocytes, appendiceal diameter	Classification of pediatric appendicitis subtypes: Negative, Uncomplicated, and Complicated	RF (Negative vs. Uncomplicated): Accuracy: 90.7% Sensitivity: 100% XGBoost (Negative vs. Complicated): Accuracy: 97.3% Sensitivity: 100%
Males et al. [24] (2024) Croatia	*n* = 551 (All underwent surgery) Pediatric Retrospective	Random Forest (Optimized for high sensitivity)	CRP, Leukocytes, Sodium, % Neutrophils, Symptom Duration, NLR.	Reduction in negative appendectomies in patients with high clinical suspicion (“High Risk”).	Sensitivity: 99.7% Specificity: 17.0% Note: Designed to avoid missing cases (false negatives), sacrificing specificity.
Gollapalli et al. [11] (2024) Saudi Arabia	*n* = 411. Retrospective study with local clinical data.	Stacking Ensemble (combining KNN, Decision Tree and Bagging)	Age, length of hospital stay (LOS), white blood cell count, neutrophil count, symptoms, migratory pain, rebound	Diagnosis and distinction between complicated and uncomplicated appendicitis; use of explainable appendicitis index (XAI)	Stacking Model: Accuracy: 92.6% Precision: 95.3% F1 Score: 92.0%
Schipper et al. [22] (2024) Netherlands	*n* = 350 Adults/General Retrospective	XGBoost (HIVE and HIVE-LAB models)	Vital signs, medical history, physical examination (McBurney’s sign, migratory pain). With and without laboratory tests.	Early triage in the emergency department. The model without a laboratory was comparable to the model with a laboratory.	AUC: 0.919 (without lab) AUC: 0.923 (with lab) It surpassed the Alvarado scale (AUC 0.824).
Phan-Mai et al. [17] (2023) Vietnam	*n* = 1950 Adults/General Retrospective	Gradient Boosting (GB) (SMOTE technique for data balancing)	Age, gender, neutrophils, CRP, ultrasound report (diameter).	Limited resource environment. Targeted screening for complicated appendicitis to prioritize surgery.	AUC: 0.894 (adjusted data) Accuracy: 82.1% Better performance than SVM and Neural Networks.
Akbulut et al. [18] (2023) Türkiye	*n* = 1797 General Retrospective	CatBoost + SHAP (Explainable AI)	Total bilirubin, WBC, Neutrophils, Ratios (NLR, WLR), CRP.	Prediction of perforated vs. non-perforated appendicitis (severity). Explainable model for clinical confidence.	AUC: 0.969 (perforated appendicitis) Sensitivity: 94.1% Specificity: 90.5% Accuracy: 92.0%
Harmantepe et al. [9] (2023) Türkiye	*n* = 345 General Retrospective	Voting Classifier (LR, KNN, SVM, ANN assembly)	Minimums: Gender only and complete blood count (WBC, Neutrophils, MPV, RDW, Platelets).	A fast and economical method for locations without access to USG/CT or to avoid radiation.	Accuracy: 86.2% Sensitivity: 83.7% Specificity: 88.6%
Martinez-Fierro et al. (2026) México (This study)	*n* = 291 (246 appendicitis, 45 controls). Retrospective cohort (Secondary level hospital).	RF for perforation detection and classification, and SVM for severity stratification	41 laboratory variables evaluated (without imaging). Sodium, platelets, MCH, MPV, INR, BUN, neutrophils	Detection and stratification in resource-limited settings without access to CT/US. Prioritizes high sensitivity for patient safety.	Detection (RF): AUC: 0.94, Sensitivity: 90%, Specificity: 100%. Perforation (RF): AUC: 0.875, Sensitivity: 100%. Severity (SVM): AUC: 0.721

ML, machine learning; RF, Random Forest; SVM, Support Vector Machine; KNN, k-Nearest Neighbors; LR, logistic regression; PAS, Pediatric Appendicitis Score; WBC, white blood cell count; MPV, mean platelet volume; RDW, red cell distribution width; CRP, C-reactive protein; INR, International Normalized Ratio; BUN, Blood Urea Nitrogen; MCH, mean corpuscular hemoglobin; LOS, length of stay; USG, ultrasonography; CT, computed tomography; AUC, area under the receiver operating characteristic curve; ANN, artificial neural network; SHAP, Shapley additive explanations; SMOTE, Synthetic Minority Over-sampling Technique; XGBoost, Extreme Gradient Boosting; AdaBoost, Adaptive Boosting; SGB, Stochastic Gradient Boosting; CART, Classification and Regression Trees; XAI, Explainable Artificial Intelligence; HIVE, Hospital Information and Vital Examination model; HIVE-LAB, Hospital Information and Vital Examination with Laboratory model.

## Data Availability

The original contributions presented in this study are included in the article. Further inquiries can be directed to the corresponding authors.

## Supplementary Materials

The following supporting information can be downloaded at: https://www.mdpi.com/article/10.3390/diagnostics16071090/s1, Table S1: Distribution and missingness of all laboratory variables prior to preprocessing and imputation.; Table S2: Final feature sets used for each machine learning task. Predictors retained after forward and backward feature selection for each predictive modeling task.

## Abbreviations

The following abbreviations are used in this manuscript:

AA	Acute Appendicitis
AdaBoost	Adaptive Boosting
AIR	Appendicitis Inflammatory Response
ANN	Artificial Neural Network
AUC	Area Under the Receiver Operating Characteristic Curve
BUN	Blood Urea Nitrogen
CART	Classification and Regression Trees
CRP	C-reactive Protein
CT	Computed Tomography
HIVE	Hospital Information and Vital Examination Model
HIVE-LAB	Hospital Information and Vital Examination with Laboratory Model
INR	International Normalized Ratio
KNN	k-Nearest Neighbors
LOS	Length of Stay
LR	Logistic Regression
MCH	Mean Corpuscular Hemoglobin
MCV	Mean Corpuscular Volume
ML	Machine Learning
MPV	Mean Platelet Volume
NLR	Neutrophil-to-Lymphocyte Ratio
PAS	Pediatric Appendicitis Score
PNR	Platelet-to-Neutrophil Ratio
PT	Prothrombin Time
RBF	Radial Basis Function
RDW	Red Cell Distribution Width
RDW-CV	Red Cell Distribution Width–Coefficient of Variation
RDW-SD	Red Cell Distribution Width–Standard Deviation
RF	Random Forest
ROC	Receiver Operating Characteristic
SGB	Stochastic Gradient Boosting
SHAP	Shapley Additive Explanations
SMOTE	Synthetic Minority Over-sampling Technique
SVM	Support Vector Machine
USG	Ultrasonography
WBC	White Blood Cell Count
WNR	White Cell-to-Neutrophil Ratio
WSES	World Society of Emergency Surgery
XGBoost	Extreme Gradient Boosting
