# Supplementary material for: Usefulness of Laboratory-Based Machine Learning for Detection and Severity Classification of Acute Appendicitis in a Resource-Limited Healthcare Setting
Source: Diagnostics (Basel). 2026 Apr 4;16(7):1090. doi: 10.3390/diagnostics16071090 (PMC13073205; doi:10.3390/diagnostics16071090)
Supplement: Supplementary file 1 [file diagnostics-16-01090-s001.zip › diagnostics-4185769-supplementary.pdf]

Usefulness of Laboratory-Based Machine Learning for Detection and Severity Classification of Acute Appendicitis in a Resource-Limited Healthcare Setting

Margarita L. Martinez-Fierro [1,†], Jose G. Gonzalez-Rodarte [1,†], Sodel Vazquez-Reyes [1,*], Manuel Gonzalez-Plascencia [1], Idalia Garza-Veloz [1], Perla Velasco-Elizondo [1], Sidere M. Zorrilla-Alfaro [1], Jaime Y. Burciaga-Paez [1], Gonzalo Ibarra-Bañuelos [1], Luis A. Flores-Chaires [1], and Alejandro Mauricio-Gonzalez [1,*]

[1] Molecular Medicine Laboratory, Academic Unit of Human Medicine and Health Sciences, Universidad Autónoma de Zacatecas, Carretera Zacatecas-Guadalajara Km.6. Ejido la Escondida, Zacatecas 98160, Mexico. margaritamf@uaz.edu.mx (M.L.M.-F.); 38192814@uaz.edu.mx (J.G.G.-R); vazquezs@uaz.edu.mx (S.V.-R.); manuelgonzalezcharro@gmail.com (M.G.P); idaliagv@uaz.edu.mx (I.G.-V.); pvelasco@uaz.edu.mx (P.V.-E.); burciagapaezjy@gmail.com (J.Y.B.-P.); gonzaloib2997@gmail.com (G.I.-B.); luischaires@uaz.edu.mx (L.A.F-CH.); amgdark@uaz.edu.mx (A.M.-G.);

† Equal contribution

* Correspondence: vazquezs@uaz.edu.mx (S.V.-R.); amgdark@uaz.edu.mx (A.M.-G.).

Supplementary Table S1. Distribution and missingness of all laboratory variables prior to preprocessing and imputation.

A. Included Variables				
**Parameter, units**	**Control group (n = 45)**		**Appendicitis group (n = 246)**	
	**Missing data n (%)**	**Value**	**Missing data n (%)**	**Value**
**Sex**				
Male, n (%)	0 (0.0)	9 (20.0)	0 (0.0)	127 (51.60)
Female, n (%)	0 (0.0)	36 (80.00)	0 (0.0)	119 (48.40)
**Age** (years)	0 (0.0)	28.36 ± 8.37	0 (0.0)	24.79 ± 19.32
**Red blood cells** ($\times 10^6$/μL)	0 (0.0)	4.76 ± 0.50	6 (2.4)	4.83 ± 0.21
**Hemoglobin** (g/dL)	0 (0.0)	13.83 ± 1.85	0 (0.0)	14.08 ± 2.10
**Hematocrit** (%)	0 (0.0)	40.30 ± 5.46	1 (0.4)	40.80 ± 4.98
**MCV** (fL)	0 (0.0)	85.89 ± 4.35	5 (2.0)	84.30 ± 1.45
**MCH** (Pg)	0 (0.0)	29.01 ± 2.21	6 (2.4)	29.07 ± 2.25
**MCHC** (g/dL)	0 (0.0)	33.50 ± 0.48	6 (2.4)	34.90 ± 0.43
**RDW-CV** (%)	1 (2.2)	12.95 ± 0.38	6 (2.4)	12.80 ± 0.26
**RDW-SD** (fL)	1 (2.2)	42.70 ± 3.38	6 (2.4)	41.15 ± 1.10
**Platelet count** ($\times 10^3$/μL)	0 (0.0)	297.09 ± 95.37	2 (0.8)	271.47 ± 80.45
**MPV** (fL)	1 (2.2)	10.70 ± 0.32	11 (4.5)	10.00 ± 0.37
**WBC count** ($\times 10^3$/μL)	0 (0.0)	9. 69 ± 3.89	3 (1.2)	14.65 ± 5.07
**Lymphocytes** ($\times 10^3$/μL)	0 (0.0)	1.82 ± 0.80	6 (2.4)	1.48 ± 0.80
**Lymphocytes (%)**	0 (0.0)	22.16 ± 12.23	4 (1.6)	8.90 ± 2.22
**Neutrophils ($\times 10^3$/μL)**	1 (0.0)	7.16 ± 3.91	10 (4.1)	11.82 ± 4.9
**Neutrophils** (%)	0 (0.0)	70.24 ± 13.98	5 (2.0)	84.30 ± 2.81
**Band neutrophils** (%)	—	Cases only	82 (33.3)	4.00 ± 2.36
**Segmented neutrophils** (%)	—	Cases only	75 (30.5)	78.00 ± 4.08
**Monocytes** (%)	—	Cases only	81 (32.9)	4.77 ± 3.15
**Prothrombin time** (Sec)	0 (0.0)	13.80 ± 0.36	19 (7.7)	15.20 ± 0.60
**aPTT** (Sec)	0 (0.0)	31.40 ± 1.10	22 (8.9)	33.40 ± 1.61
**INR** (ratio)	0 (0.0)	1.07 ± 0.03	12 (4.9)	1.23 ± 0.24
**IPL** (%)	—	Cases only	8 (3.3)	200.00 ± 38.87
**Blood group and Rh** (categorical)				
**O+**		56.1		75.6
**A+**		24.4		15
**B+**	4 (8.9)	12.2	0 (0.0)	5.3
**AB+**		4.9		2.4
**Others**		2.4		1.6

**Glucose** (mg/dL)	0 (0.0)	91.00 ± 3.83	41 (16.7)	104.00 ± 7.14
**Creatinine** (mg/dL)	0 (0.0)	0.66 ± 0.05	41 (16.7)	0.71 ± 0.08
**BUN** (mg/dL)	0 (0.0)	9.68 ± 4.40	49 (19.9)	11.38 ± 1.10
**Urea** (mg/dL)	0 (0.0)	21.16 ± 8.96	41 (16.7)	24.60 ± 2.40
**Calcium** (mg/dL)	11 (24.4)	9.15 ± 0.25	108 (43.9)	9.20 ± 0.15
**Magnesium** (mg/dL)	11 (24.4)	1.99 ± 0.07	107 (43.5)	2.00 ± 0.08
**Phosphorus** (mg/dL)	10 (22.2)	3.33 ± 0.79	108 (43.9)	3.50 ± 0.40
**Chloride (**mEq/L)	9 (20.0)	105.95 ± 1.10	86 (35.0)	101.85 ± 1.45
**Potassium** (mEq/L)	9 (20.0)	3.85 ± 0.33	85 (34.6)	3.83 ± 0.13
**Sodium** (mEq/L)	9 (20.0)	140.40 ± 1.10	85 (34.6)	137.00 ± 1.22
**Surgical diagnosis** (categorical)				
Complicated n (%)	—	Cases only	0 (0.0)	76 (30.9)
Non-complicated n (%)	—	Cases only		170 (69.1)
**Histopathological diagnosis** (categorical)				
Perforated n (%)	—	Cases only	0 (0.0)	21 (8.5)
Non-perforated n (%)	—	Cases only		225 (91.5)
**Surgical approach** (categorical)				246
Open n (%)	—	Cases only	0 (0.0)	238 (96.7)
Laparoscopic n (%)	—	Cases only		8 (3.3)
**Incision type** (categorical)				
Rocky-Davis incision, n (%)	—	Cases only	0 (0.0)	92 (37.4)
Midline incision, n (%)	—	Cases only		71 (28.9)
McBurney incision, n (%)	—	Cases only		39 (15.9)
Battle incision, n (%)	—	Cases only		36 (14.6)
Laparoscopic incision, n (%)	—	Cases only		8 (3.3)
**Stump management (**categorical)				
Pouchet technique, n (%)	—	Cases only	0 (0.0)	189 (76.8)
Zuckerman technique, n (%)	—	Cases only		32 (13.0)
Parker-Kerr technique, n (%)	—	Cases only		18 (7.3)
Halsted technique, n (%)	—	Cases only		7 (2.8)

B. Not included Variables

Parameter	Control group (n = 45)		Appendicitis group (n = 246)	
	Missing data n (%)	Value	Missing data n (%)	Value
**Total bilirubin** (mg/dL)	5 (11.1)	0.63 ± 0.21	—	Controls only
**Direct bilirubin** (mg/dL)	5 (11.1)	0.35 ± 0.20	—	Controls only
**Indirect bilirubin** (mg/dL)	5 (11.1)	0.26 ± 0.05	—	Controls only
**Albumin** (g/dL)	5 (11.1)	4.36 ± 0.13	—	Controls only
**Alkaline phosphatase** (U/L)	6 (13.3)	139.00 ± 31.75	—	Controls only
**LDH** (U/L)	5 (11.1)	391.00 ± 74.49	—	Controls only
**AST** (U/L)	5 (11.1)	32.50 ± 28.75	—	Controls only
**ALT (U/L)**	5 (11.1)	39.50 ± 75.05	—	Controls only
**Total protein** (g/dL)	5 (11.1)	6.87 ± 0.70	—	Controls only
**Lipase** (U/L)	17 (37.8)	480.00 ± 305.20	—	Controls only
**Amylase** (U/L)	17 (37.8)	369.50 ± 267.30	—	Controls only
**Creatine Kinase** (U/L)	—	Cases only	245 (99.6)	Insufficient data
**hCG assay** (mUI/mL)	—	—	246 (100.0)	—
**Serum Globulins** (g/dL)	5 (11.11)	Cases only	210 (85.4)	2.61 ± 0.55
**Procalcitonin** (ng/mL)	—	Cases only	243 (98.8)	1.40 ± 1.83
**C-Reactive Protein** (mg/L)	—	Cases only	201 (81.7)	13.65 ± 10.64
**Erythrocyte Sedimentation Rate** (mm/h)	—	*Cases only*	211 (85.8)	20.49 ± 11.43
**Length of hospital stay** (days)	0 (0.0)	5.73 ± 3.83	0 (0.0)	3.20 ± 2.82

Values are presented as mean ± standard deviation (SD). Categorical variables are reported as counts and percentages (n, %). Missing data are presented as the number and proportion of missing observations for each variable within each group. This table summarizes the statistical distribution and missingness of all laboratory variables collected in the dataset prior to preprocessing and imputation. Variables with more than 50% missing data were excluded from downstream analyses as described in the Methods section. Abbreviations: SD, standard deviation; IQR, interquartile range; n, number of observations; WBC, white blood cell count; RBC, red blood cell count; MCV, mean corpuscular volume; MCH, mean corpuscular hemoglobin; MCHC, mean corpuscular hemoglobin concentration; RDW-CV, red cell distribution width–coefficient of variation; RDW-SD, red cell distribution width–standard deviation; MPV, mean platelet volume; PT, prothrombin time; INR, international normalized ratio; aPTT, activated partial thromboplastin time; BUN, blood urea nitrogen; LDH, lactate dehydrogenase; AST, aspartate aminotransferase; ALT, alanine aminotransferase.

Supplementary Table S2. Final feature sets used for each machine learning task. Predictors retained after forward and backward feature selection for each predictive modeling task.

Predictive task	Model	Final selected predictors
**Appendicitis detection** (appendicitis vs. non-appendicitis)	Random Forest	Serum sodium, platelet count, mean corpuscular hemoglobin (MCH), mean platelet volume (MPV), international normalized ratio (INR), blood urea nitrogen (BUN), neutrophil count.
**Histopathological classification** (perforated vs. non-perforated appendicitis)	Random Forest	Mean corpuscular volume (MCV), RDW-CV, lymphocytes, segmented neutrophils, monocytes, prothrombin time (PT), INR, glucose, creatinine, potassium.
**Surgical severity stratification** (complicated vs. uncomplicated appendicitis)	Support Vector Machine (SVM)	Hemoglobin, hematocrit, RDW-SD, total leukocyte count, band neutrophils, glucose, creatinine, potassium, sodium, chloride, phosphorus, age, sex

RF, Random Forest; SVM, Support Vector Machine; MCH, mean corpuscular hemoglobin; MPV, mean platelet volume; INR, international normalized ratio; BUN, blood urea nitrogen; MCV, mean corpuscular volume; RDW-CV, red cell distribution width–coefficient of variation; RDW-SD, red cell distribution width–standard deviation; PT, prothrombin time.
